# EGFR Mutations and Tyrosine Kinase Inhibitors: Structural Insights and Therapeutic Advances

**DOI:** 10.1021/acsomega.5c11981

**Published:** 2026-02-19

**Authors:** Megha V. Manoj, Ramesh Babu Mupparaju V, Amrita Thakur, Anil Kumar Sasidharan Pillai

**Affiliations:** Department of Physical Sciences, Amrita School of Engineering, 563960 Amrita Vishwa Vidyapeetham, Bengaluru Campus, Bengaluru - 560035, India

## Abstract

Epidermal Growth Factor Receptor (EGFR) mutations are a major driver of nonsmall cell lung cancer (NSCLC), particularly among nonsmoking populations. Oncogenic mutations within the tyrosine kinase (TK) domain of EGFR play a critical role in activating downstream signaling pathways that promote tumor growth and survival. Targeting this domain has proven effective in developing therapeutic agents for NSCLC. However, treatment with these inhibitors often leads to acquired resistance due to secondary on-target mutations and activation of alternative pathways, making disease management increasingly challenging and necessitating continuous development of novel drugs and strategies. This review provides a comprehensive structural analysis of EGFR, highlighting key activating and resistance-associated mutations and their implications for drug resistance. It also examines mutation-driven resistance mechanisms and the current landscape of novel tyrosine kinase inhibitors (TKIs) in clinical development.

## Graphical abstract

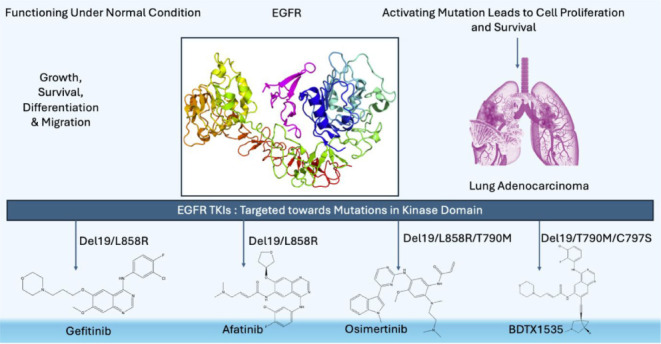

## 1 Introduction

According to estimates from 2022, lung cancer will continue to be a major global health concern, accounting for over 2.5 million new cases and 1.8 million fatalities.^1^ This substantial disease burden represents a major deterrent to achieving the United Nations Sustainable Development Goal (SDG) 3, Good Health and Well-Being for all. It is linked to cancer-related death in men and women, next to breast cancer. Nonsmall Cell Lung Cancer (NSCLC), which includes adenocarcinoma, squamous cell carcinoma, and large-cell carcinoma, constitutes 85–90% of lung cancer patients. It is associated with high mortality and a 5-year survival rate of only 18%.^2^ According to the American Lung Association, EGFR mutations are found mostly in the adenocarcinoma subtype of NSCLC. While conventional treatments remain the standard, advances in biomarker-guided and genomic research have enabled the identification of oncogenic mutations and the development of more effective, personalized therapies.^3,4^ FDA and EMA-approved TKIs, gefitinib, erlotinib, afatinib, and osimertinib, serve as first-line therapies for EGFR mutant NSCLC.^5^ However, drug resistance, adverse effects, nonspecificity, and low bioavailability limit their long-term efficacy.^6,7^ To overcome the current challenges associated with EGFR-targeted therapies, it is imperative to develop innovative strategies that address these limitations and improve long-term clinical outcomes. Recent literature on EGFR-TKIs predominantly emphasizes progress in targeted therapies, their influence on treatment paradigms, improvements in patient survival, and key findings from major clinical trials. Ciardiello et al.^8^ provided a comprehensive analysis of third-generation TKIs, outlining their development, mechanisms of inhibition, and clinical applications. In contrast, Cheng et al.^9^ conducted a bibliometric assessment to identify emerging trends in resistance to EGFR-TKIs. Additionally, Zhou et al.^10^ explored fourth-generation EGFR-TKIs, discussing design strategies, antitumor activity, binding properties, pharmacokinetic profiles, clinical progress, and prospects. A separate review^11^ traced the development of EGFR-TKIs, emphasizing promising chemical scaffolds currently progressing into phase II and III clinical trials for metastatic NSCLC with EGFR mutations. Another recent reviews^12,13^ focused on strategies to overcome secondary resistance mutations, such as T790M and C797S, providing detailed insights into EGFR signaling pathways, approved drugs, and emerging candidates. This included discussion of their chemical structures, binding interactions, efficacy, and selectivity for mutant versus wild-type EGFR. However, existing literature still lacks a comprehensive synthesis of structural insights, signaling mechanisms, mutation profiles, and the most recent advancements in TKI development, gaps that this review aims to address.

## 2 Epidermal Growth Factor Receptor

EGFR is a transmembrane glycoprotein, encoded by the EGFR gene, located at position 7p11.2 on chromosome 7. It belongs to the receptor tyrosine kinases’ ErbB family of tyrosine kinase receptors, along with proteins like the EGFR/HER1/erbB1, HER2/erbB2/neu, HER3/erb3, and HER4/erb4.^13,14^ Receptors in this family share a common architecture consisting of four key regions: an extracellular domain (EC) with two cysteine-rich regions, a single transmembrane helix, a cytoplasmic juxtamembrane (JM) domain, and an intracellular kinase (or tyrosine kinase, TK) domain, which ends with the regulatory domain in its C-terminal. Upon ligand binding and subsequent receptor activation, multiple tyrosine residues located on the C-terminal tail of this intracellular domain become phosphorylated.^15^

### 2.1 Structure of EGFR

EGFR consists of 1,210 amino acids and has an approximate molecular weight of 134 kDa.^16^ The first 24 residues, which form the N-terminal signal peptide of this protein, are generally omitted from the final structural numbering. Next is the extracellular ligand-binding domain (EC), which spans amino acid 25 to 645. It is composed of four distinct subdomains (I–IV). Within this region, subdomains I (L1) and III (L2) bind to the growth factor ligands and are characterized by a leucine-rich, β-helical structure. In contrast, subdomains II (CR1) and IV (CR2) are cysteine-rich, stabilized by disulfide bonds. CR1 facilitates both homo- and heterodimerization of EGFR with other ErbB family members.^17^ Together, L1, CR1, and L2 form a C-shaped structure that enables EGFR to bind within the groove between L1 and L2.^18^

The transmembrane region is a 22-amino acid segment (residues 646–668) forming a hydrophobic α-helical peptide that spans the membrane and connects the extracellular and intracellular domains. It is essential for the allosteric modulation of EGFR, which occurs through the pivoting and rotational motion of its helices.^19^ These dynamics are directly influenced by the surrounding lipid environment, as the thickness of the bilayer can affect the stability of certain conformations and ultimately control receptor activation.^20–22^ The C-end of the transmembrane (TM) region is connected to the intracellular kinase domain via the JM domain. The JM domain is essential for receptor dimerization and activation.^23^ Although relatively short (∼37 amino acids), the JM domain harbors multiple functional elements, including lysosomal and basolateral sorting motifs,^24^ a nuclear localization signal,^25^ a calmodulin-binding site,^26^ and phosphorylation sites for protein kinase C,^27^ and MAPK.^28^ Phosphorylation at Thr654 reduces the domain’s ability to regulate receptor activation while its mutation enhances kinase phosphorylation.^23^ These domains are presented in Figure 1, section A. The TK domain (residues 645–674) follows the JM domain. It consists of a relatively smaller N-terminal lobe (N-lobe) and a larger C-terminal lobe (C-lobe). The N-lobe contains five β-strands (β1−β5) and an αC-helix (residues 729–744), while the C-lobe includes five α-helices (αE−αI). The kinase domain features an ATP-binding site within a cleft between its two lobes, located beneath a conserved glycine-rich loop that connects the N-lobe’s β1 and β2 strands^29^ and coordinates ATP phosphates via backbone interactions.^30^ The C-lobe also contains the conserved catalytic loop (Asp812–Asn818), where Asp812 interacts with the substrate’s hydroxyl (−OH) group and Asn818 stabilizes its orientation through hydrogen bonding. A key regulatory element within the C-lobe is the activation loop (A-loop), spanning residues Asp831–Val852. The base of this loop is anchored by a highly conserved Asp-Phe-Gly (DFG) motif, essential for controlling kinase catalytic activity.^26^ Figure 1B illustrates the structure of the kinase domain, based on the crystal structure with PDB ID 2ITX.

**Figure 1:**
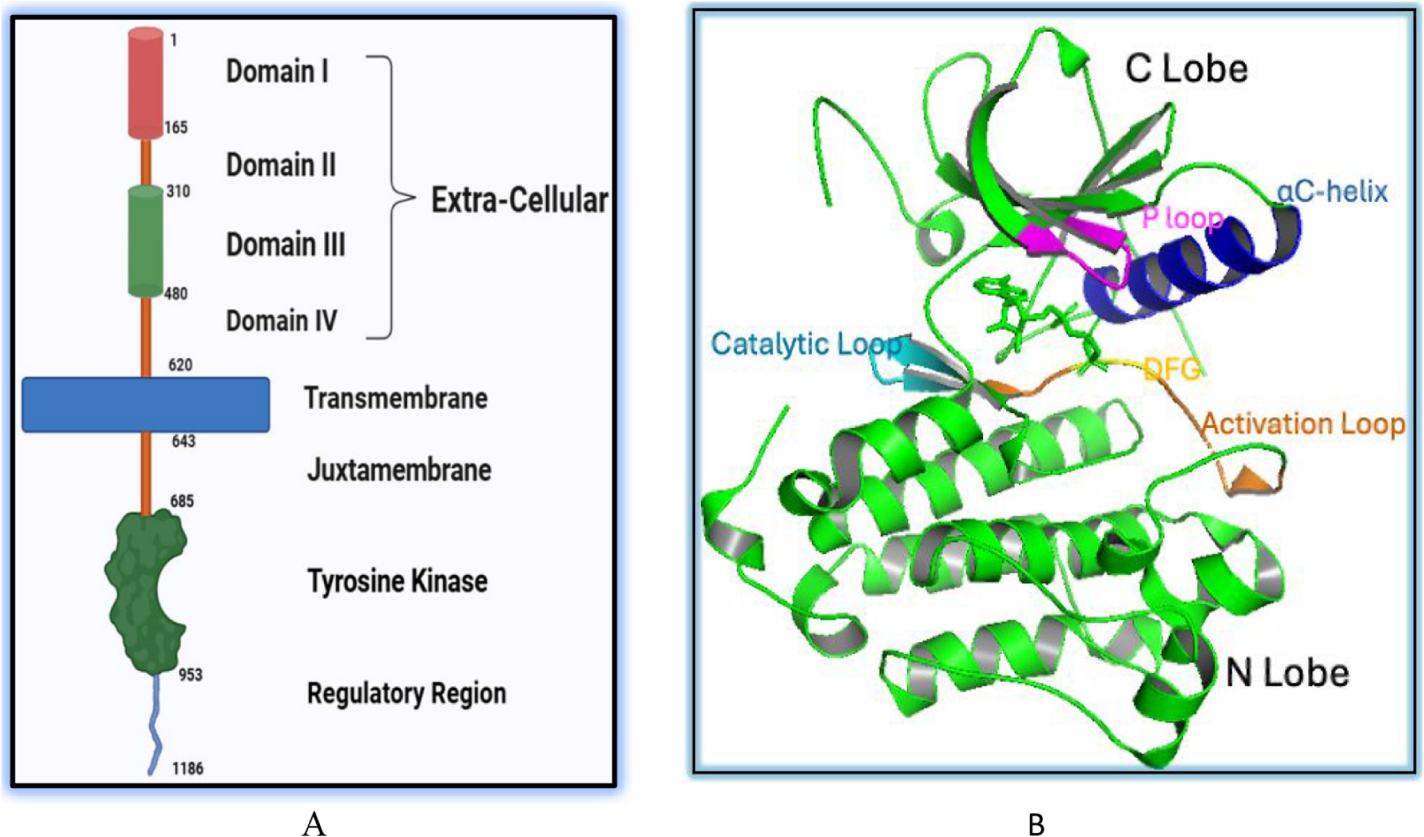
(A) The domain structure of the Epidermal Growth Factor Receptor (EGFR). The receptor is composed of an extracellular region, containing the L1, CR1, L2, and CR2 subdomains. The intracellular portion includes the juxtamembrane (JM) and the tyrosine kinase (TK) domain. TK domain is responsible for catalysis and ATP binding, and the C-terminal regulatory domain controls the kinase’s activity. (B) Crystal structure of the EGFR tyrosine kinase (TK) domain (PDB ID: 2ITX) shown in complex with the ATP analog, AMP-PNP, visualized using PyMOL (Schrödinger, LLC).

EGFR ends with the C-terminal regulatory domain, comprising 229 amino acids (residues 982–1210). In the absence of autophosphorylation, it plays a critical role in inhibiting kinase activity.^18,31^ Its proline-rich region contains multiple phosphorylation sites. The proximal segment near the kinase domain contributes to autoinhibition and has been structurally characterized. An α-helix motif, known as the AP-2 helix, encompassing residues 997–1001 and mediates interaction with the clathrin-associated adaptor protein 2 (AP-2) complex.^32,33^ This helix stabilizes an inactive dimer configuration by engaging with the N-lobe of the neighboring TK domain.^34,35^ Following the AP-2 helix is an acidic segment termed the “electrostatic hook” (residues 1003–1022), which interacts with the kinase’s hinge region to mediate inhibitory dimerization via electrostatic forces; this interaction ends upon phosphorylation.^33^ The hook’s C-terminal portion forms a β-strand that inhibits JM latch formation. The critical role of this region in kinase activation is evident, as deletion of Tyr1210 (within the NPXY motif) significantly reduces phosphorylation at Tyr869 in the kinase domain.^32^

### 2.2 “EGFR-Mediated” Activation of Downstream Signaling Pathways

The protein exists as an inactive monomer within the plasma membrane. Activation of unmutated wild-type (WT) EGFR depends on the binding of ligands such as epidermal growth factor (EGF) or transforming growth factor-alpha (TGF-α).^36^ In its inactive state, the extracellular domain (EC) adopts a compact, tethered conformation, with subdomain II interacting with subdomain IV. This interaction conceals the dimerization arm (a β-hairpin loop in subdomain II), thereby preventing spontaneous dimerization.

The A-loop within the TK domain remains in a closed conformation, blocking substrate access and maintaining an autoinhibited structure that prevents ATP binding and phosphorylation. Upon ligand binding between subdomains I and III, these domains move closer together, stabilizing the ligand-binding pocket and disrupting the tether between subdomains II and IV. This conformational rearrangement exposes the dimerization arm, enabling EGFR to dimerize with another activated EGFR monomer.^37,38^ Within the dimer, one TK domain acts as the activator and the other as the receiver. The activator’s kinase domain interacts asymmetrically with the JM segment of the receiver kinase. This allosteric activation leads to phosphorylation of tyrosine residues in the regulatory domain, inducing conformational changes required for kinase activation and subsequent phosphorylation.^39^ The EGFR activation process is illustrated in Figure 2. Phosphorylated tyrosine serves as docking sites for intracellular adaptor proteins, initiating major downstream signaling pathways such as RAS/RAF/MEK/ERK and PI3K/AKT. These pathways regulate essential cellular functions, including growth, survival, differentiation, and migration^40^ as shown in Figure 3.

**Figure 2:**
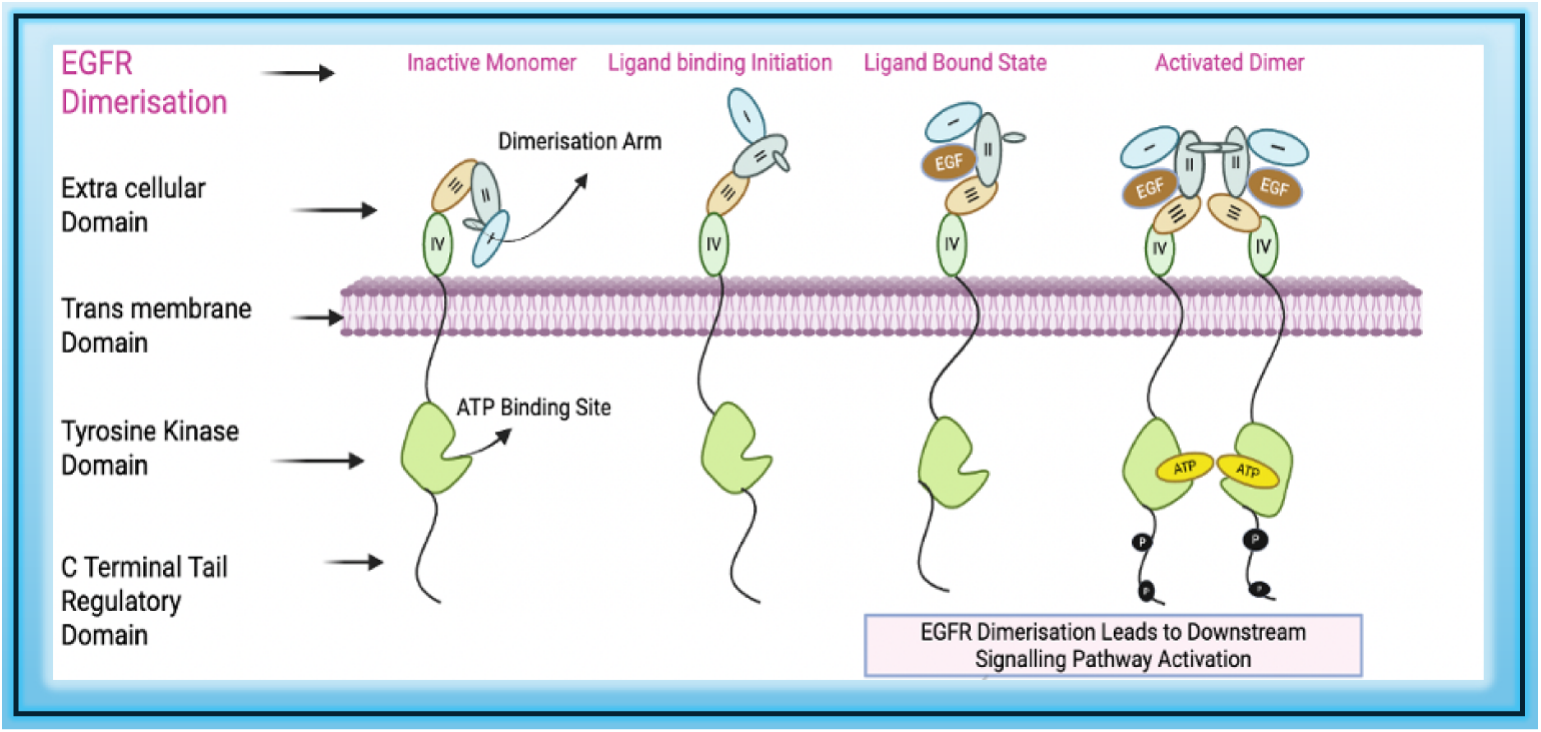
EGFR activation begins with ligand binding in the extracellular domain leading to its dimerization, ATP binding and phosphorylation if tyrosine residue in the regulatory region.

**Figure 3:**
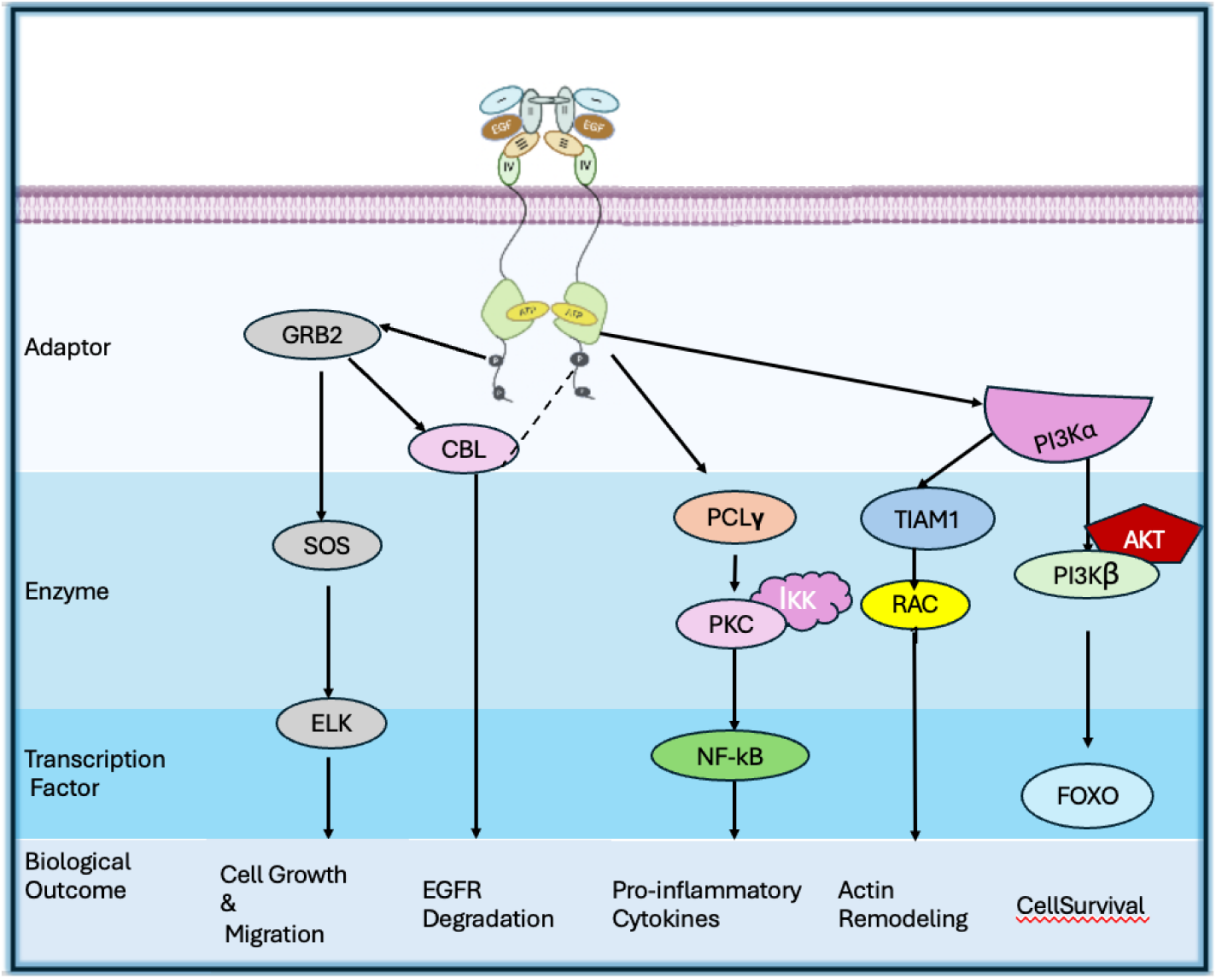
EGFR activation pathways “Adapted from Uribe, M. L., Marrocco, I., & Yarden, Y. (2021). EGFR in cancer: Signaling mechanisms, drugs, and acquired resistance. Cancers, 13(11), 2748.”^40^

Under normal conditions, phosphorylation of WT EGFR triggers receptor internalization, followed by ubiquitination and degradation, processes that tightly regulate EGFR signaling. EGFR levels are controlled through endocytosis, directing the receptor either toward lysosomal degradation for permanent inactivation or recycling back to the plasma membrane for reuse.^41^ Overexpression of EGFR and its ligands is a key factor in the development of cancers in the lung, breast, and colorectal regions.^42^ In contrast to WT EGFR, mutant EGFRs not only exhibit hyperphosphorylation in the absence of ligand,^43,44^ but also resist downregulation and degradation.^45^ Study by Chen et al., have shown that in NSCLC-cell lines, mutant EGFR expression correlates with reduced levels of key negative regulators, such as the tumor suppressor CD82. While WT EGFR promotes CD82 expression, mutant forms suppress it, suggesting that CD82 downregulation may facilitate EGFR overexpression and enhance the tumorigenic potential driven by EGFR mutations.^46^

### 2.3 Structural Changes in the Kinase Domain during Activation and ATP Binding

The TK domain, like other kinases, can switch between active and inactive states, with its structures well-characterized in the Protein Data Bank (PDB). Active and inactive monomeric kinase conformations are primarily distinguished by the positioning of the αC-helix and the activation loop (A-loop). Inactive EGFR’s kinase domain exists in two main conformations: the Src-like and the DFG-out states. The Src-like conformation, observed in PDB structures, is defined by the outward movement of the αC-helix (“αC-out”) and disruption of the critical glutamate–lysine salt bridge.^47^ A key feature of this state is a small hydrophobic helix at the N-terminal end of the A-loop, which directly interacts with the αC-helix. Notably, the kinase maintains a DFG-in orientation in this form. In some inactive structures, the DFG-out conformation has also been observed, characterized by a flipped DFG motif where the aspartate points away from the ATP-binding site and the A-loop is extended. The αC-helix in this state partially overlaps with that of the Src-like conformation. Activation occurs through structural rearrangements in the intracellular kinase domain, including repositioning of the N-lobe and C-lobe, which opens the ATP-binding cleft.^48^

The activator subunit pushes the receiver subunit’s αC-helix inward, into the active conformation. This aligns key residues Glu762 and Lys745 to form a Glu–Lys salt bridge, enabling ATP binding. In the active conformation, Glu738 present in the αC-helix forms a stabilizing ion pair with Lys721 from β3. The Lys721 also bonds with ATP’s phosphate groups. These structural transitions are essential for the regulation of kinase’s activity. Additionally, the A-loop, which contains the conserved DFG motif, adopts an open, extended structure with the catalytic aspartate oriented toward the ATP-binding pocket (“DFG-in” conformation).^49^ The structural transition shifts EGFR into its catalytically active form, capable of substrate binding, phosphorylation and downstream signaling. This conformational transition from the inactive to the active state creates space within the kinase domain, allowing ATP to bind. The A-loop, previously blocking the substrate-binding site, shifts into an open conformation, exposing the ATP-binding cleft, and the ATP gains access to the binding site.^47,50^ Figure 4 highlights the structural differences between the active (PDB ID: 2ITP) and inactive Src-like (PDB ID: 2GS7) kinase forms in the key structural domains of the EGFR.

**Figure 4:**
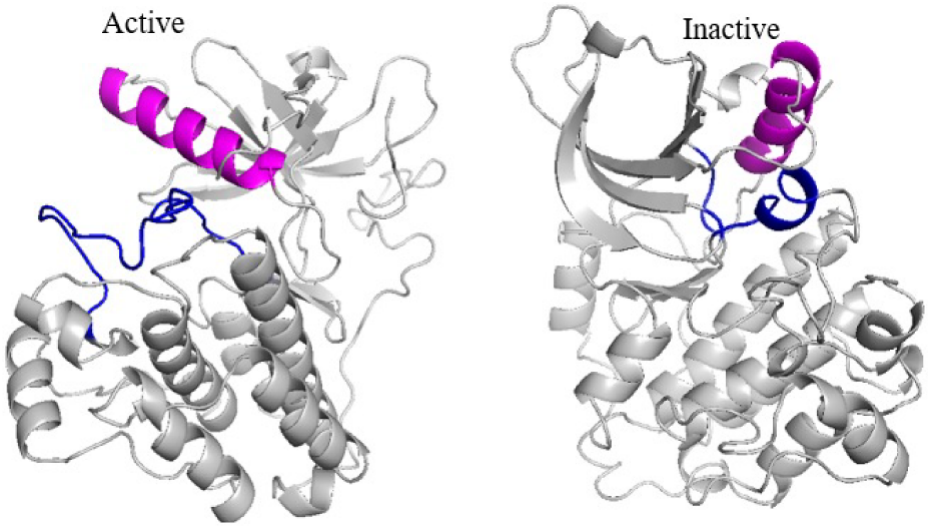
Structural comparison of the active and inactive states of the EGFR kinase domain (PDB IDs: 2ITX) for active and 2GS7 for inactive. The conformational transition to the active state repositions the activation loop, opening the ATP-binding cleft and enabling ATP access. visualized using PyMOL (Schrödinger, LLC).

Critical H-bond interactions occur between ATP and the hinge region of the kinase domain, specifically involving residues Gln791 and Met793. These hydrogen bonds stabilize ATP binding within the cleft, positioning it for catalysis. Once ATP is securely bound, the γ-phosphate group is transferred to tyrosine residues on the C-terminal tail of EGFR. Oncogenic EGFR mutations modify EGFR’s structure, keeping it constitutively active, even without ligand binding. This results in spontaneous dimerization, ATP binding, and autophosphorylation of key intracellular tyrosine residues.^51^ The following section discusses EGFR mutations observed in NSCLC patients, their impact on EGFR activation, and therapeutic significance.

**Figure 5:**
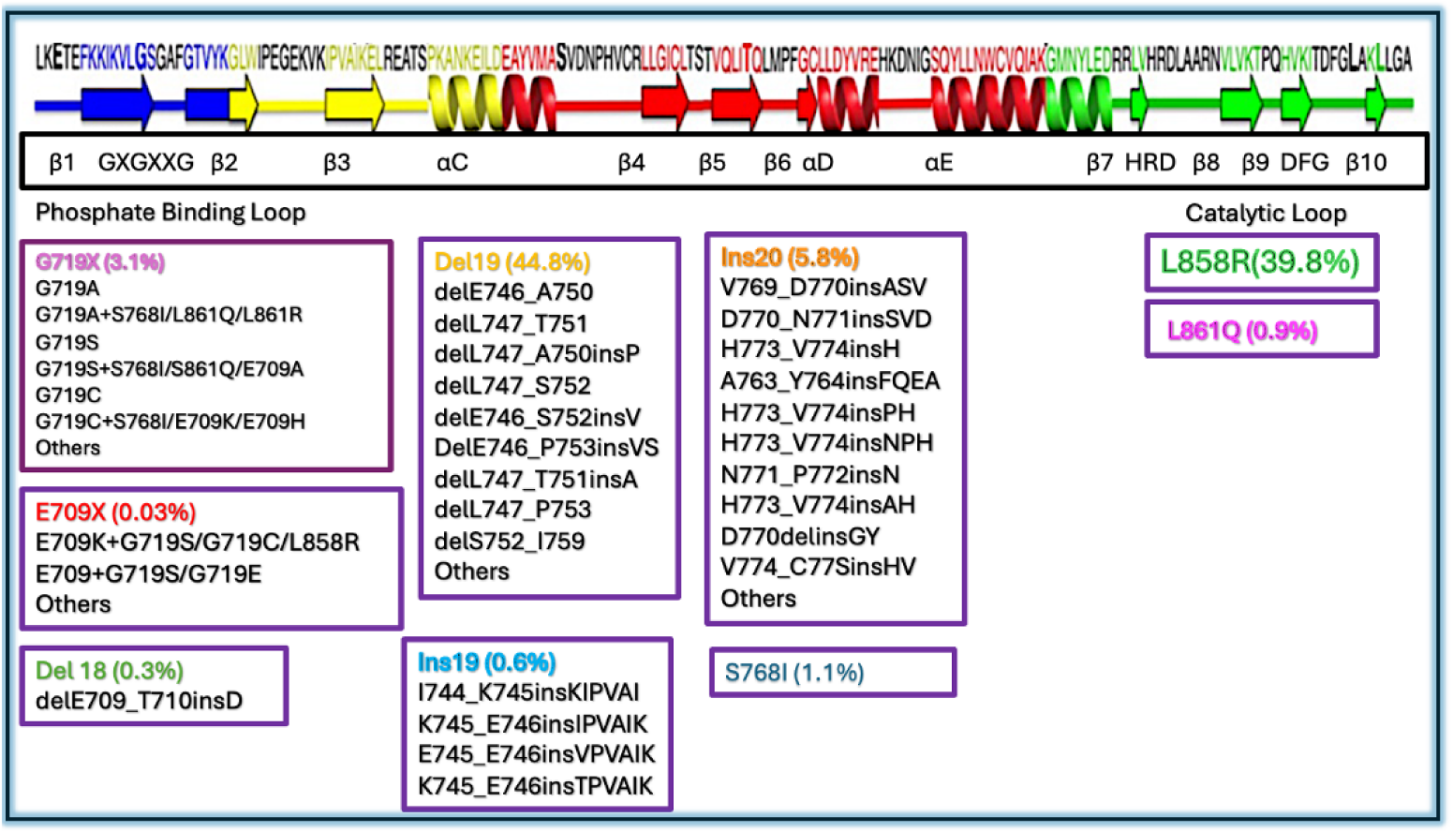
Epidermal growth factor receptor (EGFR) protein showing mutations associated with lung cancer. Representative mutation frequencies are mapped to codons in exons 18, 19, 20, and 21 (highlighted in blue, yellow, red, and green, respectively), with structural elements indicated as α-helices (spiral motifs) and β-sheets (thick arrows). Individual amino acid residues are represented using single-letter notation. Adapted from Kobayashi, Y., and Mitsudomi, T. (2016). Not all epidermal growth factor receptor mutations in lung cancer are created equal: perspectives for individualized treatment strategy. Cancer Science, 107(9), 1179–1186.^55^

## 3 Oncogenic EGFR Mutations in NSCLC

Somatic EGFR mutations in NSCLC cells were first identified in 2004.^52^ The prevalence and spectrum of these mutations vary significantly across ethnic populations, with reported frequencies of 10–15% in European and North American groups, approximately 26% in Latin American populations, and up to 62% in Asian populations.^53^ Oncogenic EGFR mutations alter the receptor’s conformation, keeping it constitutively active even without ligand binding. This results in ligand-independent dimerization and/or spontaneous autophosphorylation of key intracellular tyrosine residues. Such uncontrolled activation not only drives cancer cell survival but also enables the tumor microenvironment to shield the tumor, promoting resistance to therapy and inhibiting programmed cell death.^54^

Kobayashi et al. and Zhang Y. L.^55^ compiled data from several large-scale studies (Figure 5), including the Catalog of Somatic Mutations in Cancer (COSMIC), which incorporates next-generation sequencing (NGS) data and mutation-specific diagnostic kits. COSMIC reports over 590 distinct EGFR mutation types, with 93% located in exons 18–21 encoding the TK domain.^56^

EGFR mutations are broadly classified as classical or nonclassical based on their response to TKIs.

### 3.1 Classical Mutations

Classical EGFR mutations account for 80–90% of EGFR-sensitive NSCLC cases.^55^ These activating mutations occur in exons 18–21 of the EGFR gene, which encode the TK domain, and are considered TKI-sensitizing. The most common classical mutations include exon 19 deletions (ex19del) and the Leu858Arg (L858R) point mutation in exon 21. The L858R mutation, located within the A-loop of the TK domain, represents approximately 41% of EGFR mutations in NSCLC. Exon 19 encodes part of the TK domain, specifically the β3 strand and αC-helix, spanning residues Glu746–Ala750 near the substrate-binding site. Approximately 44% of EGFR mutations involve ex19del, with delGlu746_Ala750 being the most frequent subtype, followed by delLeu747_Pro753insSer, delLeu747_Ala750insPro, and delLeu747_Thr751insPro.^57^ These deletions remove a conserved sequence critical for maintaining the inactive kinase conformation, causing the αC-helix to shift inward and stabilize the active form. Consequently, EGFR remains constitutively active, even without ligand binding, resulting in persistent autophosphorylation and continuous activation of downstream signaling pathways.^58–60^ This structural change has also been found to disrupts the hydrophobic environment that stabilizes the inactive state, further promoting activation.^61^

Classical mutations drive uncontrolled cell proliferation and tumor growth, contributing to NSCLC pathogenesis. Importantly, the L858R mutation exhibits high sensitivity to EGFR TKIs because the altered ATP-binding pocket Favors drug binding.^62^ Figure 6 illustrates classical and resistant mutations within the kinase domain.

**Figure 6:**
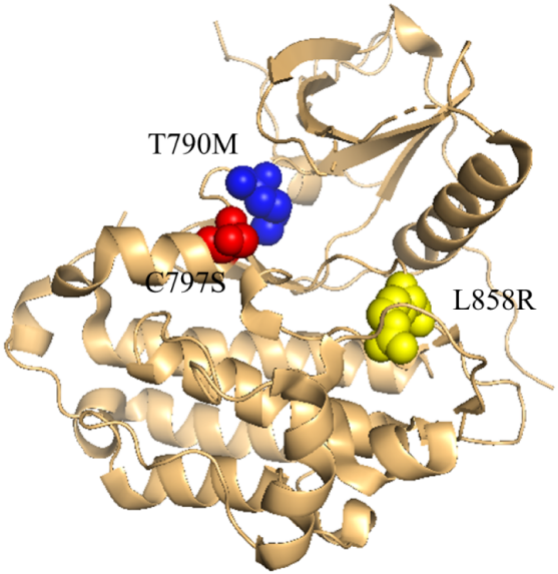
Structure of EGFR highlighting key point mutations. The L858R substitution represents a primary activating mutation, while T790M and C797S are common secondary mutations associated with acquired drug resistance (PDB ID: 6LUB), visualized using PyMOL (Schrödinger, LLC).

### 3.2 Nonclassical Mutations

Nonclassical mutations represent approximately 12% of EGFR alterations and are less common, often underrepresented in clinical studies. Some NSCLC tumor harbor multiple EGFR mutations simultaneously. Nonclassical mutations exhibit variable sensitivity to TKIs, making their clinical significance heterogeneous. Clinically actionable subsets include insertions in exon 19 (ins19), deletions in exon 18 (del18), and point mutations such as Glu709X (E709X).^63,64^

Exon 19 insertions are rare, accounting for only 0.41% of EGFR mutations. He et al. identified an 18-base pair insertion causing a substitution of Pro for Leu at residue 747. In-frame ex20ins, observed in 3–7% of NSCLC cases, typically involve 3–21 base pair insertions or duplications between amino acids 761–775. These alterations activate the receptor independently of ligand binding. More than 60 unique insertions, mostly 1–4 amino acids in the loop following the αC-helix, have been reported, showing significant heterogeneity compared to del19 mutation.^65^

Rare point mutations include Gly719Ter (G719X), del18, E709K, Glu709X, Ala767_Val769dup, Ser768Ile, and Leu861Gln. G719X mutations in exon 18 involve substitutions at position 719 and may exhibit partial TKI sensitivity depending on the variant.^66^ Another rare mutation, S768I/V in exon 20, frequently co-occurs with other EGFR mutations, with variable TKI responses. E709X mutations in exon 18 also demonstrate diverse inhibitor sensitivity. Frameshift mutations, though rare (0.91% of cases), can confer resistance to standard TKIs, necessitating alternative therapeutic strategies.^67–69^ The heterogeneity of these mutations underscores the need for further research to develop effective treatments for these rare variants.

## 4 ATP and Allosteric Binding Sites for EGFR TKIs in NSCLC

TKIs act through two primary mechanisms:

(i) competing with ATP at the substrate-binding pocket, and
(ii) binding to allosteric sites.

The ATP-binding site of EGFR comprises five distinct regions: the adenine-binding region, sugar (ribose-binding) region, hydrophobic region I, hydrophobic region II, and the phosphate-binding region.^70^ Most currently available EGFR inhibitors function as ATP-competitive small molecules, specifically designed to interact with the adenine-binding region and the two hydrophobic regions.^71,72^ These inhibitors share critical pharmacophoric features for optimal binding. A planar heteroaromatic ring system enables TKIs to compete with ATP in the adenine-binding pocket and form hydrogen bonds with key residues such as Met769, Thr790, and Thr854.^73^ Additionally, a hydrophobic tail engages hydrophobic region I through multiple nonpolar interactions and often extends into hydrophobic region II, enhancing overall binding affinity.^74,75^

The allosteric site is located within the inner hinge region of the TK domain, adjacent to the αC-helix and beneath the ATP-binding cleft, between the N-lobe and C-lobe of the kinase domain.^76,77^

Structurally, the allosteric pocket includes a hydrophobic channel near both the A-loop and phosphate-binding loop (P-loop), positioned beneath the hinge region and adjacent flexible segments. The αC-helix plays a pivotal role in enabling inhibitor-induced conformational changes.^78^ Effective allosteric inhibitors incorporate key pharmacophoric features: a hydrophobic anchor group to engage the buried groove, a hinge-avoiding scaffold to maintain non-ATP-competitive interaction, and functional groups capable of forming hydrogen bonds or π–π stacking with residues such as Met766, Leu858, and Asp855.^79,80^ The two binding pockets, with inhibitors EAI045 and osimertinib bound at the allosteric and ATP binding site respectively, are shown in Figure 7.

**Figure 7:**
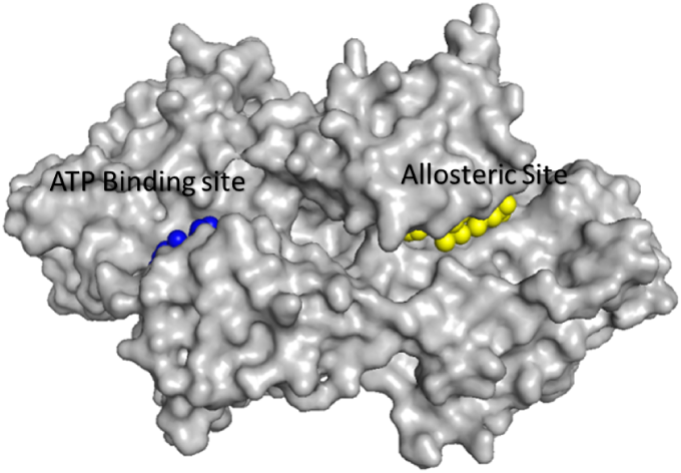
Locations of the ATP and allosteric binding sites. Close-up view illustrating the allosteric inhibitor EAI045 (yellow spheres) bound near the αC-helix and the osimertinib (blue spheres) occupying the ATP binding pocket. Rendered from PDB ID: 7JXM, visualized using PyMOL (Schrödinger, LLC).

Due to conserved structure and unstable hydrogen bonding, the ATP-binding pocket is challenging to target selectively, often resulting in off-target effects and resistance. Inhibitors that target allosteric site overcome these limitations by stabilizing EGFR in its inactive state and preventing ATP binding. Notably, they can bypass resistance mutations such as T790M and C797S, offering a valuable therapeutic alternative in NSCLC.^80–82^ Their enhanced selectivity also helps reduce off-target toxicity.

## 5 EGFR Tyrosine Kinase Inhibitors

EGFR TKIs are classified into generations based on their development timeline, ability to overcome resistance mutations, and interaction with mutant EGFR.^83^

First-generation TKIs inhibit mutated EGFR in its active DFG-in conformation by competing with ATP. Their 4-aminoquinazoline core forms a hydrogen bond with Met769 in the hinge region of the binding pocket. Additional structural features, such as hydrophobic rings, solvent-accessible side chains, and an −NH– linker, confer specificity and flexibility. FDA-approved first-generation drugs, gefitinib, erlotinib, and icotinib, demonstrate comparable adjuvant efficacy in early stage EGFR mutant NSCLC, achieving objective response rates of 50–80% in treatment-naïve advanced disease and prolonging progression-free survival in classical EGFR mutations. However, resistance typically develops within 9–15 months due to the T790M mutation in exon 20, which reduces drug binding.^84–87^

Second-generation TKIs, including FDA-approved afatinib, neratinib, and dacomitinib, retain the anilino-quinazoline scaffold but incorporate an acrylamide moiety for irreversible covalent binding to Cys797.^88^ While dacomitinib demonstrated superior progression-free survival compared to gefitinib, afatinib showed limited activity against T790M, and most second-generation agents were associated with dose-limiting skin and gastrointestinal toxicities due to wild-type EGFR inhibition.^89,90^

Third-generation TKIs selectively inhibit T790M-mutated EGFR while sparing wild-type EGFR, minimizing toxicity. These inhibitors feature acrylamide warheads on aminopyrimidine scaffolds for covalent bonding with Cys797 and incorporate structural optimizations for improved potency, selectivity, pharmacokinetics, and brain penetration.^91,92^ The binding mechanism of these inhibitors with representative drugs gefitinib, dacomitinib and osimertinib is shown in Figure 8.

**Figure 8:**
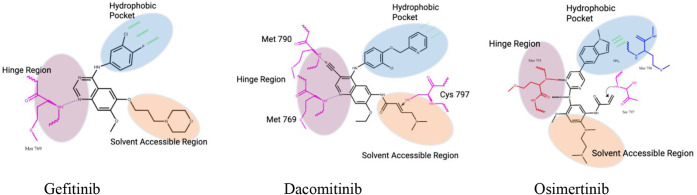
First-generation TKIs (e.g., gefitinib) contain an aminoquinazoline core that mimics the adenosine moiety of ATP and form a hydrogen bond with Met769 in the hinge region. To overcome the T790M mutation, second-generation TKIs (e.g., dacomitinib) incorporate an acrylamide group for covalent bonding with Cys797. Third-generation TKIs (e.g., osimertinib) feature an aminopyrimidine scaffold, selectively targeting the T790M resistance mutation while minimizing toxicity-associated adverse effects. Adapted from Mansour, M. A., Aboul Magd, A. M., Abbas, S. H., Abdel-Rahman, H. M., and Abdel-Aziz, M. (2023). Insights into fourth generation selective inhibitors of (C797S) EGFR mutation combating nonsmall cell lung cancer resistance: a critical review. RSC Advances, 13(27), 18825–18853.^92^

Osimertinib (Tagrisso), an FDA-approved third-generation EGFR-TKI, demonstrated superior progression-free survival compared to platinum-based chemotherapy in patients with T790M-positive, locally advanced or metastatic NSCLC in the phase III AURA3 trial (NCT02151981).^93^ It is currently being evaluated in the phase III LAURA study (NCT03521154; active, not recruiting), for its efficacy and safety following chemoradiation in patients with unresectable stage III NSCLC harboring EGFR-sensitizing mutations (Ex19Del, L858R), alone or in combination with other EGFR alterations.^94,95^ Osimertinib’s design incorporates an acrylamide group for covalent binding to Cys797, a pyrimidine core to mimic ATP, and an aniline substituent for enhanced selectivity. It also forms hydrogen bonds with Met793 in the hinge region, providing greater spatial hindrance at the ATP-binding site in mutant EGFR compared to the wild type.^96^

Several third-generation EGFR-TKIs have obtained regulatory approvals for the treatment of NSCLC from the U.S. FDA, the National Medical Products Administration (NMPA), China, and the Medicines and Healthcare products Regulatory Agency (MHRA), UK. Some of the approved third-generation TKIs are shown in Figure 9, while Table 1 presents the details of these TKIs.

**Figure 9:**
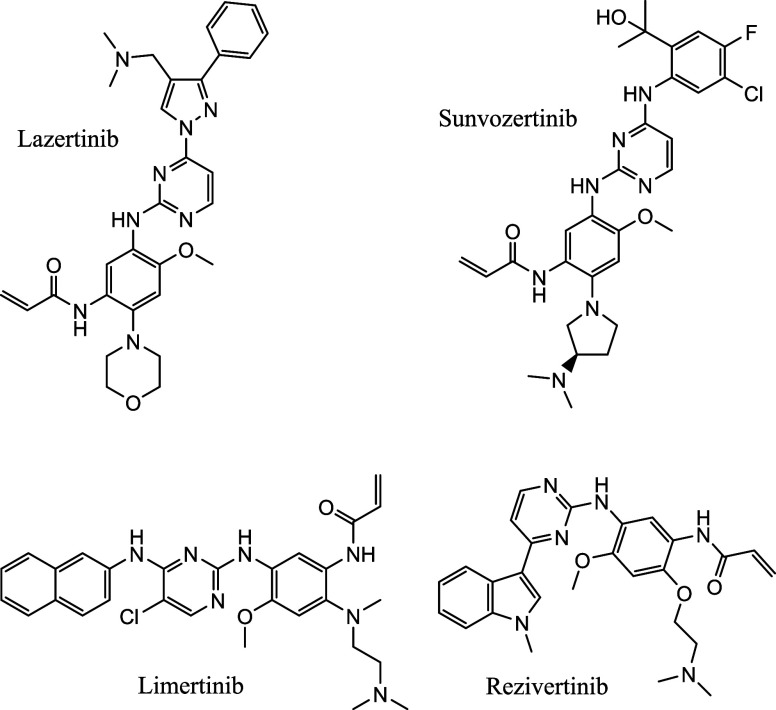
Approved third generation TKIs.

**Table 1:** Approved Third Generation TKIs

**Drug and Developer**	**Clinical Trial Status**	**NCT Identifier**
Lazertinib (Janssen Biotech, Inc.)	FDA granted approval based on the phase III MARIPOSA trial (2024) to be used with amivantamab as first-line drug for locally advanced metastatic mutation in patients harboring exon19del and exon 21 L858R substitution mutations.^97^	NCT04487080
Firmonertinib (Allist Pharmaceuticals)	It received first approval (NMPA) for the treatment of patients with locally advanced or metastatic NSCLC with confirmed T790M mutation and disease has progressed during or after EGFR TKI therapy.^98,99^ The global, registrational Phase 3 FURVENT (FURMO-004) trial, a randomized, multicenter, open-label study, is ongoing to evaluate patients with locally advanced or metastatic nonsquamous NSCLC harboring EGFR exon 20 insertion mutations who have not received prior treatment.^98^	NCT05607550
Aumolertinib (HS-10296) (Hansoh Pharmaceutical Group Co, Ltd.)	Approved in China (2020) for the treatment of advanced NSCLC harboring T790M mutations based on the results of the APOLLO trial,^100^ A subsequent Phase III single-arm, open-label study^101^ was initiated to evaluate its efficacy and safety of as neoadjuvant and adjuvant therapy for patients having potentially resectable stage III EGFR- sensitive NSCLC.^102^ Most recently, on June 3, 2025, the MHRA approved it for adult patients with locally advanced or metastatic NSCLC harboring T790M mutation-positive NSCLC.^103^	NCT02981108
		NCT04685070
Limertinib (Jiangsu Osaikang Pharmaceutical Co. and ASK Pharm.)	Approval by the NMPA (2025) for the treatment of adults with locally advanced or metastatic NSCLC harboring T790M resistant mutation, and also as a first-line treatment for NSCLC with EGFR ex19del or L858R mutations.^104^ A phase II clinical trial has been registered to evaluate its combination with carboplatin and etoposide in EGFR mutant NSCLC with small-cell lung cancer (SCLC) transformation following TKI progression.^105^	NCT07001995
Oritinib (SH-1028) (Nanjing Sanhome Pharmaceutical)	Approved by the NMPA in June 2024 for the treatment of T790M-mutated stage IV metastatic NSCLC.^106^ A Phase II open-label study, initiated in 2019, evaluated the safety and efficacy of oritinib in patients with resistant T790M mutations.^107^ Additionally, a Phase III randomized, double-blind trial comparing oritinib with gefitinib as first-line therapy for EGFR-sensitizing mutations has completed enrollment, further supporting its clinical development trajectory.^108^	NCT03823807
		NCT04239833
Rezivertinib (BPI-7711) (Beta Pharma)	Approved by the NMPA on December 29, 2024, for first-line treatment of EGFR-mutated NSCLC, including patients with brain metastases.^109^ It is effective against T790M, L858R, and exon 19 deletions, with an improved toxicity profile. The ongoing REZOR Phase III trial, a multicenter, double-blind, randomized study, is comparing rezivertinib with gefitinib in patients with locally advanced or metastatic disease across 50 hospitals in China.^110^	NCT03866499
Sunvozertinib Dizal Pharmaceutical China	The U.S. FDA granted accelerated approval in July 2025 for Sunvozertinib to treat adult patients with locally advanced or metastatic NSCLC harboring EGFR exon 20 insertion mutations who had progressed after platinum-based chemotherapy.^111^ This approval was based on results from the multinational, open-label Phase II WU-KONG1b trial and followed the drug’s Breakthrough Therapy designation. The NMPA had previously approved the drug in 2023.^112^	NCT03974022

Osimertinib therapy frequently results in acquired drug resistance over time, driven by both on-target and off-target mechanisms. Among on-target mutations, C797S is the most common and clinically significant contributor to resistance. This mutation involves the substitution of cysteine (Cys797) with serine (Ser797) within the ATP-binding pocket, which disrupts covalent bonding with the acrylamide warhead of osimertinib (Figure 10), thereby reducing binding affinity and therapeutic efficacy.^113^ Niederst et al. (2015)^114^ demonstrated that treatment response is influenced by the allelic configuration of C797S relative to T790M. When these mutations occur in trans, tumors may retain partial sensitivity to combined first- and third-generation TKIs, whereas cis configurations confer complete resistance to both monotherapy and combination regimens. Additionally, approximately 84% of resistant cases exhibit concurrent off-target mechanisms, including EGFR amplification (48%), MET amplification (16%), PIK3CA mutations (15%), and BRAF V600E mutations (5%).^115^

**Figure 10:**
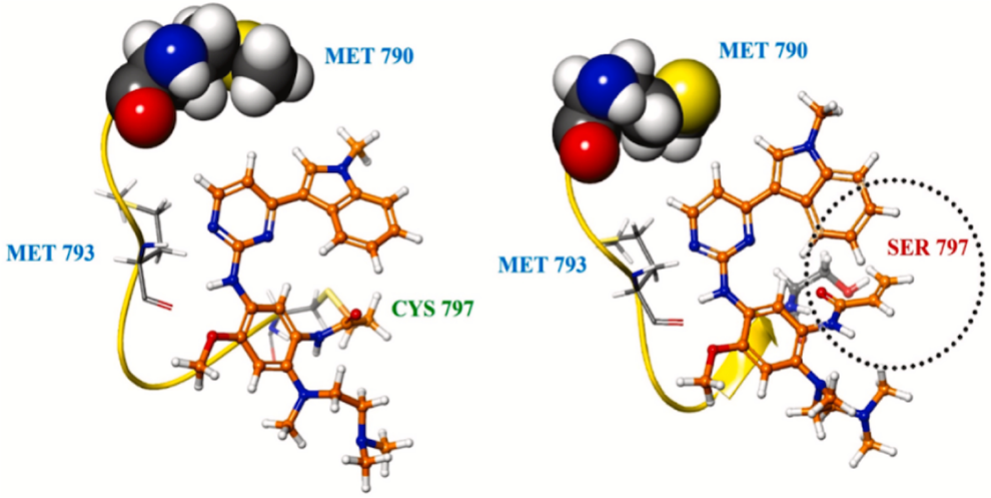
Mechanism of resistance to osimertinib caused by Cys797→Ser797 substitution in the ATP-binding pocket, preventing covalent interaction with the acrylamide warhead. Reproduced from J. Med. Chem. 2025, American Chemical Society. License Number: 6182310240808.^113^

The clinical challenges posed by EGFR mutation heterogeneity, the rising incidence of brain metastases, and the emergence of resistance following osimertinib therapy underscore the urgent need for developing additional third-generation TKIs. Several irreversible EGFR and pan-ERBB inhibitors are currently under active evaluation for their efficacy and safety in advanced NSCLC, with representative examples shown in Figure 11.

**Figure 11:**
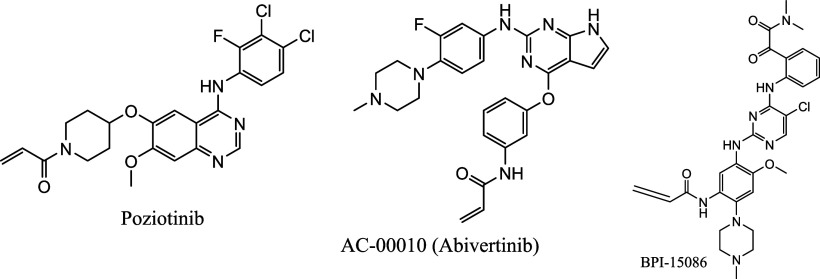
Some of the approved third generation TKIs.

Poziotinib, an oral quinazoline-based pan-ERBB inhibitor targeting HER2 exon 20 insertions, has demonstrated clinical activity; however, it was denied FDA approval for previously treated advanced NSCLC patients.^116^ Abivertinib (AC0010), a third-generation EGFR inhibitor with limited blood–brain barrier penetration, has shown promising results in Phase I/II trials conducted across China, the United States, and Europe, and has received FDA clearance to initiate a Phase II study in osimertinib-resistant NSCLC.^117^ BPI-15086, another third-generation EGFR-TKI with specificity for EGFR and T790M mutations, remains under investigation following completion of Phase I studies.^118,119^ Similarly, Futibatinib, an oral covalent EGFR-TKI with broad activity against L858R, T790M, HER2, and HER4 mutations, has exhibited early clinical efficacy in T790M-positive NSCLC, with manageable toxicities including thrombocytopenia, rash, fever, and interstitial lung disease.^120,121^ The clinical trial status and mutation profiles of these advanced EGFR and pan-ErbB1 inhibitors are summarized in Table 2.

**Table 2:** Third Generation TKIs in Clinical Trials

**Drug and Developer**	**Clinical Trial Status**	**NCT Identifier**
Poziotinib Hanmi Pharmaceutical, China	Poziotinib achieved an objective response rate of 27.8%, a disease control rate of 70%, and a median progression-free survival (PFS) of 5.5 months in patients with EGFR/HER2 ex20ins in the phase II ZENITH20 trial^116^ However, based on these results, the FDA Oncologic Drugs Advisory Committee (ODAC) voted against approval, concluding that the potential risks outweighed the benefits.	NCT03318939
AC-00010 (Abivertinib) Hangzhou ACEA Pharmaceutical Research,China	Abivertinib (AC0010) is a dual EGFR and BTK TKI with immunomodulatory properties through suppression of pro-inflammatory cytokines. It is currently under investigation for NSCLC and B-cell malignancies. The FDA has granted IND clearance for a Phase II study to evaluate its safety, pharmacokinetics, and preliminary efficacy in patients with EGFR T790M-positive, osimertinib-resistant NSCLC.^117^	NCT02330367
BPI-15086 Betta Pharmaceuticals China	A Phase I study evaluated the safety, tolerability, and pharmacokinetic profile of BPI-15086 in patients with T790M-resistant mutations.^118^ The trial also assessed its antitumor activity and explored biomarkers associated with therapeutic efficacy; however, the study was later terminated.^119^	NCT02914990
Futibatinib (TAS-12) Taiho Pharmaceutical Co. Japan	TAS-121 demonstrated promising antitumor activity in T790M-positive patients, with adverse effects including dermatologic toxicity, thrombocytopenia, and pyrexia in the Phase I study.^120^ It has also been assessed in Phase I/II trials in advanced solid tumors to determine its maximum tolerated dose and efficacy in cancers harboring FGF/FGFR aberrations, such as cholangiocarcinoma and other FGFR-driven malignancies.^121^	JapicCTI-142651
		NCT02052778

### 5.1 Fourth Generation TKI

Fourth-generation EGFR-TKIs were developed to overcome the C797S resistance mutation. These agents target the ATP-binding pocket, the allosteric pocket, or both. ATP-competitive inhibitors interact with key hinge region residues such as Lys745 and Asp800, mimicking ATP binding.^11,93^ Figure 12A illustrates BI-4020, an ATP-competitive inhibitor in clinical trials, bound to the triple mutant T790M/C797S/V948R EGFR (PDB ID: 7KXZ). In contrast, non-ATP-competitive allosteric inhibitors have shown success against C797S-mediated resistance. Structural studies reveal that allosteric inhibitors of EGFR T790M adopt a distinctive Y-shaped configuration, featuring two hydrophobic aromatic groups and an amido-isothiazole moiety for critical receptor interactions.^122^ Figure 12B depicts EA1045 bound to the triple mutant C797S/T790M/V948R (PDB ID: 5ZWJ). To address limitations of ATP-competitive and conventional allosteric inhibitors, a novel class of ortho-allosteric drugs has emerged. These dual-site binders engage both the ATP-binding and allosteric pockets, potentially eliminating the need for combination therapy with monoclonal antibodies such as cetuximab.^123^

**Figure 12:**
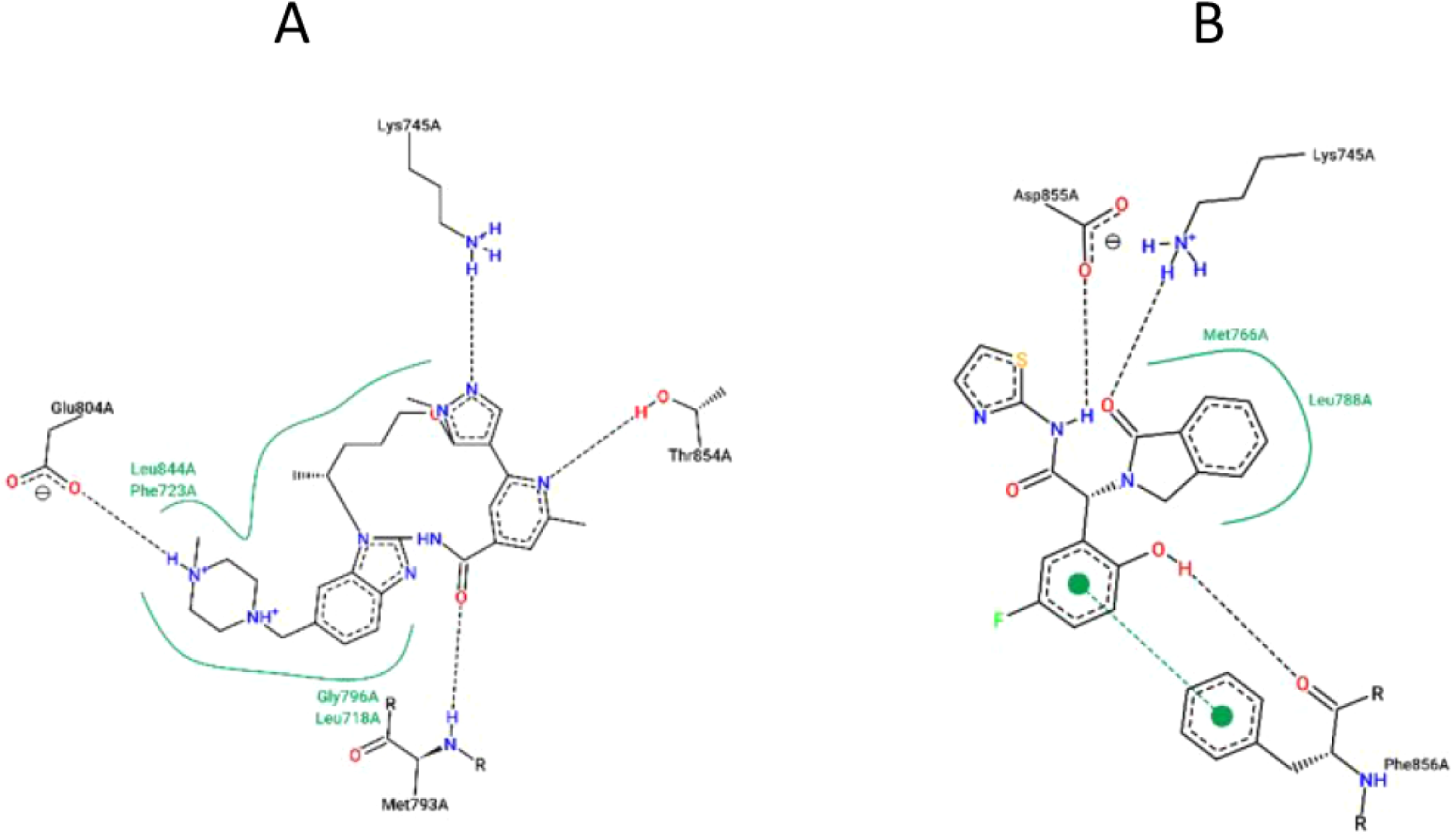
(A) BI-4020 binding in the ATP binding pocket retrieved from PDB ID: 7KXZ. (B) EA1045 an allosteric inhibitor bonded with triple mutant C797S/T790M/V948R retrieved from PDB ID: 5ZWJ. visualized using Proteins Plus.

Most fourth-generation EGFR-TKIs are in Phase I/II clinical development. Silevertinib (BDTX-1535), an orally available, irreversible, brain-penetrant EGFR inhibitor, selectively targets Exon 18–21 mutations, including C797S, Lys718Gln, and EGFRvIII, with notable CNS penetration (Kpuu ∼ 0.5).^124,125^ Phase I enrollments are complete, and a Phase II trial involving 43 patients with classical/nonclassical driver mutations or acquired C797S mutation is ongoing.^126^ BBT-207, a reversible EGFR-TKI, demonstrates preclinical potency against Lys792His, T790M, and C797S mutations and favorable CNS distribution. Its open-label, multicenter Phase I/II trial is assessing safety, tolerability, pharmacokinetics, pharmacodynamics, and preliminary antitumor efficacy through dose escalation and expansion.^127^ H002, another oral EGFR inhibitor, offers strong mutant selectivity, high oral bioavailability, brain penetration, and minimal off-target effects. A Phase I/IIa study is evaluating its safety, tolerability, pharmacokinetics, and antitumor activity in locally advanced or metastatic NSCLC harboring active EGFR mutations.^128^

Other early stage candidates include JIN-A02 (Phase I/II) and QLH11811 (Phase I in the U.S. and China) for resistant NSCLC mutations. JIN-A02 trials comprise three parts: determining maximum tolerated dose (MTD) in patients with C797S or T790M mutations, assessing safety and Phase II dose, and expanding dosing in EGFR mutant patients poststandard therapy.^129^ QLH11811 Phase I study evaluates safety, tolerability, and preliminary efficacy through dose escalation (1a) and cohort expansion (1b).^130^ BPI-361175 exhibits dose-dependent EGFR phosphorylation inhibition with high mutant selectivity and CNS activity.^131^ TQB-3804 inhibits triple mutations (EGFR del746–750/T790M/C797S) and suppresses signaling in resistant models.^132,133^ HS-10375 targets EGFR C797S and is under Phase I/II evaluation for safety, tolerability, pharmacokinetics, and monotherapy efficacy in resistant NSCLC.^134,135^ PH009-1 (ESPH009-1) shows potent preclinical efficacy, minimal wild-type activity, and strong tumor regression; recruitment has not yet started.^136^

Several next-generation inhibitors, including BLU-945, TAS3351, and BBT-176, entered early clinical trials for resistant NSCLC harboring EGFR T790M and C797S mutations. Despite promising preclinical profiles, mutant selectivity, wild-type sparing, and strong CNS penetration, these trials were discontinued. BLU-945 showed early efficacy but was terminated due to limited clinical benefit.^137^ Similarly, the Phase I/II study of WJ13405 for C797X mutations was terminated in 2023 following a strategic R&D adjustment.^138^ TAS3351 demonstrated robust brain activity but faced clinical translation challenges.^140^ BLU701.^141^ and BBT-176 exhibited strong preclinical potency and early tumor regression signals; however, development was halted due to insufficient efficacy or potential toxicity concerns.^142^ Details of these trials are summarized in Table 3.

**Table 3:** Status of Fourth Generation TKI in Clinical Trial

**Drug and Developer**	**Clinical Trial Status**	**NCT Identifier**
Silevertinib (BDTX-1535) Black Diamond Therapeutics	Phase 1/2 conducting on patients with nonclassical or acquired C797S mutation with or without the CNS or glioblastoma expressing EGFR mutations.^125^ (Active, not recruiting) Another phase 2 clinical trial is also ongoing.^126^	NCT05256290
BBT-207 Bridge Biotherapeutics, Inc.	Phase 1/2 for patients with advanced NSCLC has shown preclinical efficacy against L792H, T790M, and C797S mutations.(Active, not recruiting).^127^	NCT05920135
H002 RedCloud Bio	Phase 1/2 a patients with locally advanced or metastatic NSCLC patients. (Recruiting)^128^	NCT05519293
JIN-A02 Nanjing Hongyun Biotechnology Co.	Phase 1/2 to determine maximum tolerable dose, safety and dosing in patients harboring T790M and C797S mutation. (Recruiting)^129^	NCT05394831
QLH11811 Qilu Pharmaceutical Co., Ltd.	Phase 1 study to determine the safety, tolerability, and preliminary efficacy.(Recruiting)^130^	NCT05555212
BPI-361175 Betta Pharmaceuticals Co., Ltd.	Phase 1/2 planned for dose escalation and dose expansion (Not yet recruiting)^131^	NCT05329298
TQB-3804	Shown inhibition of triple mutations (EGFR d746–750/T790M/C797S) and suppression of signaling pathway in resistant models.^132^ The clinical trial status is currently unknown.^133^	NCT04128085
HS-10375 Jiangsu Hansoh Pharmaceutical Co.	Phase 1/2, to evaluate safety, Tolerability, Pharmacokinetics, and Efficacy for Monotherapy for C797S mutated NSCLC patients. (Recruiting).^135^	NCT05435248
PH009-1 Suzhou Puhe Pharmaceutical Technology Co., LTD	Phase 1 dose escalation and expansion planned.(Not yet recruiting).^136^	NCT06590194
BLU-945 Blueprint Medicines Corp.	Phase 1/2, Phase 1 study planned to evaluate the pharmacokinetic, pharmacodynamic, safety and tolerability and anticancer activity as monotherapy or in combination with osimertinib (SYMPHONY). Sponsor decided to terminate, not based on safety concern.^138^	NCT04862780
WJ13404 Suzhou Junjing BioSciencesCo.	Phase 1/2 clinical trial for dose expansion, efficacy expansion, safety, tolerability and PK evaluation for patients with C797X mutation. Terminated due to adjustment in R&D strategy.^139^	NCT05662670
TAS-3351 Taiho Pharmaceutical Co., Ltd.	Phase 1/2, Terminated, Strategic decision, not based on safety concerns.^140^	NCT05765734
BLU-701 Zai Lab (Shanghai) Co., Ltd.	Phase1 study (HARMONY) to evaluate the drug for monotherapy, or in combination with osimertinib or Pt based chemotherapy was conducted. Drug terminated due to lack of efficacy.^141^	NCT05153408
BBT-176 Bridge Biotherapeutics, Inc.	Phase 1/2, in patients with advanced NSCLC, Sponsor decided to terminate citing the changing landscape of NSCLC treatment.^142^	NCT04820023

Some of the above-mentioned fourth generation TKIs, with known structures are shown in Figure 13.

**Figure 13:**
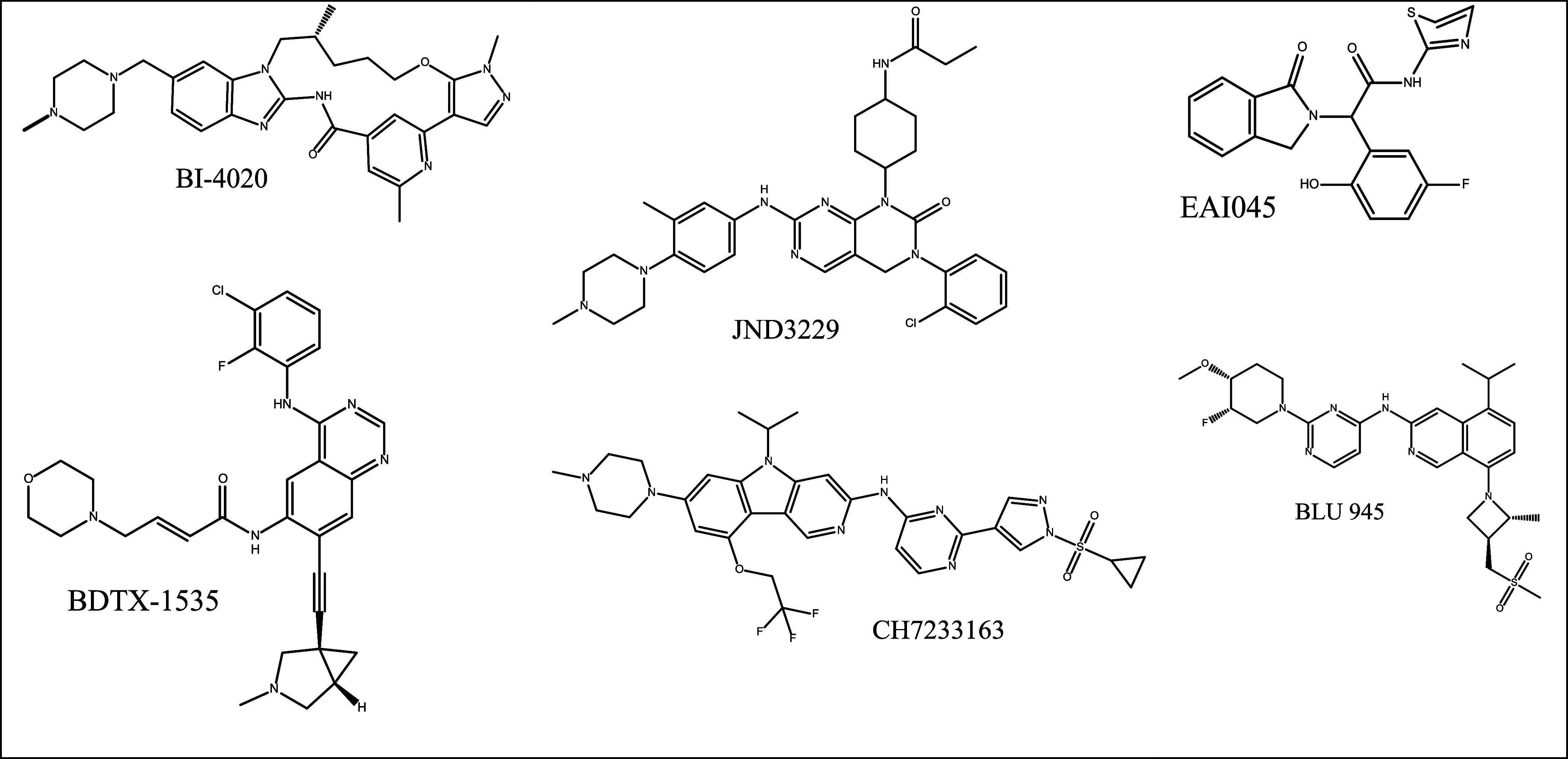
Fourth generation TKIs in different stages of clinical trial.

## 6 Challenges in EGFR TKI Development and Future Scope

EGFR mutations significantly influence TKI development in lung adenocarcinoma by determining efficacy, resistance patterns, and therapeutic strategies. Classical activating mutations ex19del and L858R enabled first-generation TKIs (gefitinib, erlotinib), while resistance from T790M and toxicity of second-generation TKIs led to third-generation inhibitors like osimertinib with covalent binding. The heterogeneity of EGFR mutations and the emergence of drug resistance make TKI development highly challenging. Fourth-generation TKIs, including ATP-competitive, allosteric, and ortho-allosteric inhibitors, seek to overcome resistance through alternative binding modes; however, issues related to potency, selectivity, and pharmacokinetics persist.

The C797S mutation remains the most critical on-target resistance to osimertinib, while alternative pathway activation, such as HER2 and MET amplification, PIK3CA mutations, PTEN loss, IGF-1R signaling, and oncogenic fusions (RET, BRAF, FGFR3, NTRK1, ALK), further undermines efficacy. Phenotypic changes like NSCLC-to-SCLC transformation and EMT add complexity. Although allosteric inhibitors offer promise by bypassing C797 interaction, their limited ability to fully inhibit EGFR dimers and potential toxicity in combination regimens restrict clinical success. These challenges underscore the need for next-generation strategies, including multitargeted approaches and optimized inhibitor designs, to sustain therapeutic benefit.

### 6.1 Future Scope

Looking ahead, EGFR-targeted therapies hold strong potential for advancement. Innovations in AI-driven drug design and structural biology will enable the development of highly selective inhibitors, addressing tumor heterogeneity, drug resistance, and off-target toxicity. Emerging strategies such as dual-target inhibitors and targeted protein degradation may further overcome resistance and expand treatment options. Additionally, combination approaches integrating TKIs with immunotherapy, chemotherapy, and other modalities are expected to play a pivotal role in shaping future therapeutic landscapes.

### 6.2 Declaration of Generative AI and AI-Assisted Technologies in the Writing Process

During the preparation of this manuscript, the authors used Copilot and Grammarly to improve clarity, accuracy, and overall language quality. After using these tools, the authors reviewed and edited the content as needed and take full responsibility for the final version of the manuscript.

## Data Availability

This article is a review and does not contain any new experimental data. All data discussed in this manuscript are from previously published sources, which are properly cited within the text.
